# Association between alcohol consumption and the risk of gastric cancer: a meta-analysis of prospective cohort studies

**DOI:** 10.18632/oncotarget.20880

**Published:** 2017-09-14

**Authors:** Zheng He, Ting-Ting Zhao, Hui-Mian Xu, Zhen-Ning Wang, Ying-Ying Xu, Yong-Xi Song, Zhong-Ran Ni, Hao Xu, Song-Cheng Yin, Xing-Yu Liu, Zhi-Feng Miao

**Affiliations:** ^1^ Department of Radiation Oncology, First Hospital of China Medical University, Shenyang, Liaoning Province, China; ^2^ Department of Breast Surgery, First Hospital of China Medical University, Shenyang, Liaoning Province, China; ^3^ Department of Surgical Oncology, First Hospital of China Medical University, Shenyang, Liaoning Province, China; ^4^ School of Life Science, Faculty of Science, University of Technology, Sydney, Australia; ^5^ Department of Medical Oncology, Shengjing Hospital of China Medical University, Shenyang, Liaoning Province, China

**Keywords:** gastric cancer, alcohol consumption, meta-analysis, heavy alcohol consumption, cancer risk

## Abstract

Alcohol consumption is inconsistently associated with the risk of gastric cancer morbidity and mortality. The aim of this study was to systematically evaluate the association between alcohol consumption on gastric cancer risk. The PubMed, Embase, and Cochrane Library databases were searched from inception through April 2017. Prospective cohort studies evaluating the association between alcohol consumption and risk of gastric cancer which report its effect estimates with 95% confidence intervals (CIs) were included. The results summary was performed using the random-effect model. Twenty-two cohort studies involving 22,545 cases of gastric cancer and 5,820,431 participants were identified and included in our data analysis. Overall, drinking had little or no effect on gastric cancer as compared with non-drinkers. Furthermore, light and moderate alcohol consumption had no significant effect on gastric cancer risk when compared with non-drinkers. However, heavy alcohol consumption was associated with a greater risk of gastric cancer when compared with non-drinkers. The findings of the subgroup analyses indicated that light alcohol consumption was associated with a lower risk of gastric cancer in women, while heavy alcohol consumption was associated with an increased risk of gastric cancer regardless of country, gender, whether the study reported gastric cancer incidence, or whether the study adjusted for body mass index, educational attainment, or physical activity. The findings of this study suggest that light alcohol consumption might play a protective effect on gastric cancer in women, while heavy alcohol consumption is associated with a significantly increased risk of gastric cancer in all subgroups.

## INTRODUCTION

Gastric cancer is the fourth most common form of cancer and the second most common cause of cancer-related deaths in the world, despite the declining incidence and mortality rates in recent decades [1]. The incidence of gastric cancer approximated 988,602 new cases and 723,000 deaths in 2013 worldwide [2]. Several environmental and lifestyle factors have already been established to play important roles in the etiology of gastric cancer [3–13]. Several meta-analyses have illustrated the effect of alcohol consumption on subsequent gastric cancer incidence and mortality [14, 15], however, whether these relationships differ according to different participant characteristics remains controversial.

The mechanism of the relationship between alcohol consumption and gastric cancer risk remains unclear. Recent evidence suggests that the high-density lipoprotein levels and anti-inflammatory effects associated with alcohol consumption might play an important role on the progression of gastric cancer [16]. Furthermore, the effect of alcohol abuse has already been established, with increased alcohol consumption associating with an increased risk of certain cancers. Previous studies have also evaluated the relationship between alcohol consumption and gastric cancer risk, yet these studies report inconclusive results [17–38]. Clarifying the optimal daily intake of alcohol is particularly important for the general population, as it has not been definitively determined. To obtain a more comprehensive understanding of the association between alcohol consumption and gastric cancer risk, we conducted a meta-analysis of prospective cohort studies to systematically evaluate the association between alcohol consumption and the risk of gastric cancer incidence or mortality and to compare these associations among participants with different baseline characteristics.

## RESULTS

### Literature search

**Figure 1:**
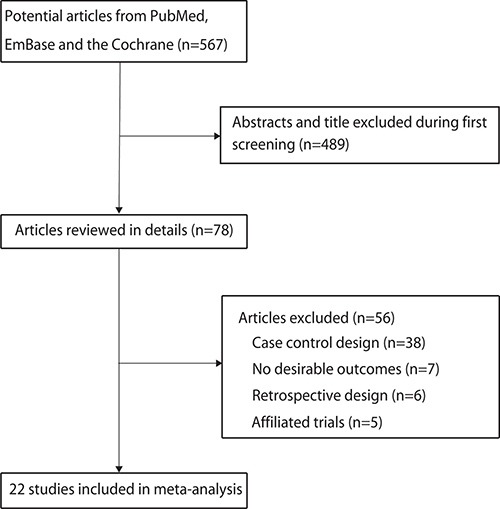
Study selection process

The results of the study-selection process are shown in Figure 1. We identified 567 articles in our initial electronic search, of which 489 were excluded as duplicates and irrelevant studies, leaving a total of 78 potentially eligible studies for further evaluation. After detailed evaluations, 22 prospective cohort studies were selected for the final meta-analysis [17–38]. A manual search of the reference lists of these studies did not yield any new eligible studies. The general characteristics of the included studies are presented in Table 1.

**Table 1:** Baseline characteristics of studies included in the systematic review and meta-analysis

Study	Country	Sample size	Age at baseline	Gender (M/F)	GC incidence/ death cases	Follow-up (year)	Adjusted factors	NOS score
Gordon 1984 [17]	US	5209	29–62	2106/2641	18	22.0	Age, SBP, cigarettes/day, relative weight, and lipoproteins	7
Kono 1987 [18]	Japan	5130	NA	5130/0	116	19.0	Age, smoking	6
Stemmermann 1990 [19]	US	7572	≥ 45	7572/0	174	22.0	Age, smoking	6
Kato 1992 [20]	Japan	9753	≥ 30	NA	57	6.0	Sex, age, smoking, cooking methods, family history of stomach cancer	7
Galanis 1998 [21]	US	5546	≥ 18	5546/0	64	14.8	Age, education, place of birth, smoking	7
Fujino 2002 [22]	Japan	44930	≥ 18	18746/26184	379	7.3	Age	8
Sasazuki 2002 [23]	Japan	19657	40-59	19657/0	293	10.0	Age, area, smoking, fruit, vegetable, salted cod roe or fish gut intake, BMI	8
Barstad 2005 [24]	Denmark	28463	21-93	15236/13227	122	13.7	Sex, age, smoking	7
Nakaya 2005 [25]	Japan	21201	40–64	21201/0	247	7.2	Age, smoking, education, orange, other fruit juice, spinach, carrot or pumpkin and	7
Study	Country	Sample size	Age at baseline	Gender (M/F)	GC incidence/ death cases	Follow-up (year)	Adjusted factors	NOS score
							tomato consumption	
Larsson 2006 [26]	Sweden	61433	40-76	0/61433	160	15.7	Age, education, vegetable, fruit, processed meat and coffee intake, smoking	8
Sjodahl 2006 [27]	Norway	69962	≥ 15	34202/35760	251	16.0	Sex, age, BMI, education, smoking	8
Freedman 2007 [28]	US	474606	≥ 50	282856/191750	472	4.6	Sex, age, BMI, education, physical activity, vegetable, fruit and energy intake, smoking	8
Sung 2007 [29]	Korea	669570	≥ 30	669570/0	3452	6.5	Age, BMI, smoking, preference for saltiness in food	7
Kim 2010 [30]	Korea	2248129	30–80	1420981/827148	12393	6.7	Age, sex, BMI, smoking habits, physical activity, and family history of cancer	7
Steevens 2010 [31]	Netherlands	3962	55–70	1944/2018	655	16.3	Sex, age, smoking, BMI, education, energy, fruit, vegetable and fish intake	7
Moy 2010 [32]	China	18244	45–64	18244/0	391	20.0	Education, BMI, smoking, and summed intakes of preserved food items, fresh fruits, and fresh vegetables	8
Study	Country	Sample size	Age at baseline	Gender (M/F)	GC incidence/ death cases	Follow-up (year)	Adjusted factors	NOS score
Kim 2010 [33]	Korea	1341393	40–69	919199/422194	1326	5.0	Age, residential, physical activity, BMI, SBP, DBP, and fasting blood sugar	7
Duell 2011 [34]	Europe	478459	35–70	142601/335858	444	8.7	Age, sex, center, education, smoking, and intake of fruit/nuts/seeds, vegetables, processed and red meat, and total energy	8
Everatt 2012 [35]	Lithuania	7150	40–59	7150/0	185	30.0	Smoking, education level and BMI	7
Yang 2012 [36]	China	218189	40–79	218189/0	1137	15.0	Age, area, smoking and education	8
Jung 2012 [37]	Korea	16320	≥ 20	6405/9915	93	9.3	Age, sex, BMI, smoking habit, geographic area, and educational attainment	7
Jayalekshmi 2015 [38]	India	65553	30–84	65553/0	116	8.0	Age, calendar time, occupation, education, and smoking	7

*GC: gastric cancer; SBP: systolic blood pressure; DBP: diastolic blood pressure; BMI: body mass index.

### Study characteristics

The twenty-two prospective cohort studies involved a total of 5,820,431 participants and data on 22,545 cases of gastric cancer morbidity or mortality. The follow-up period for participants was 4.6–30.0 years, while 3,962–2,248,129 individuals were included in each study. Four studies were conducted in the US [17, 19, 21, 28], 6 in Europe [24, 26, 27, 31, 34, 35], and the remaining 12 in Asia [18, 20, 22, 23, 25, 29, 30, 32, 33, 36–38]. Study quality was evaluated using the NOS. Overall, 8 studies had a score of 8 [22, 23, 26–28, 32, 34, 36], 12 studies had a score of 7 [17, 20, 21, 24, 25, 29–31, 33, 35, 37, 38], and the remaining 2 studies had a score of 6 [18, 19].

### Drinking versus non-drinking for gastric cancer

**Figure 2:**
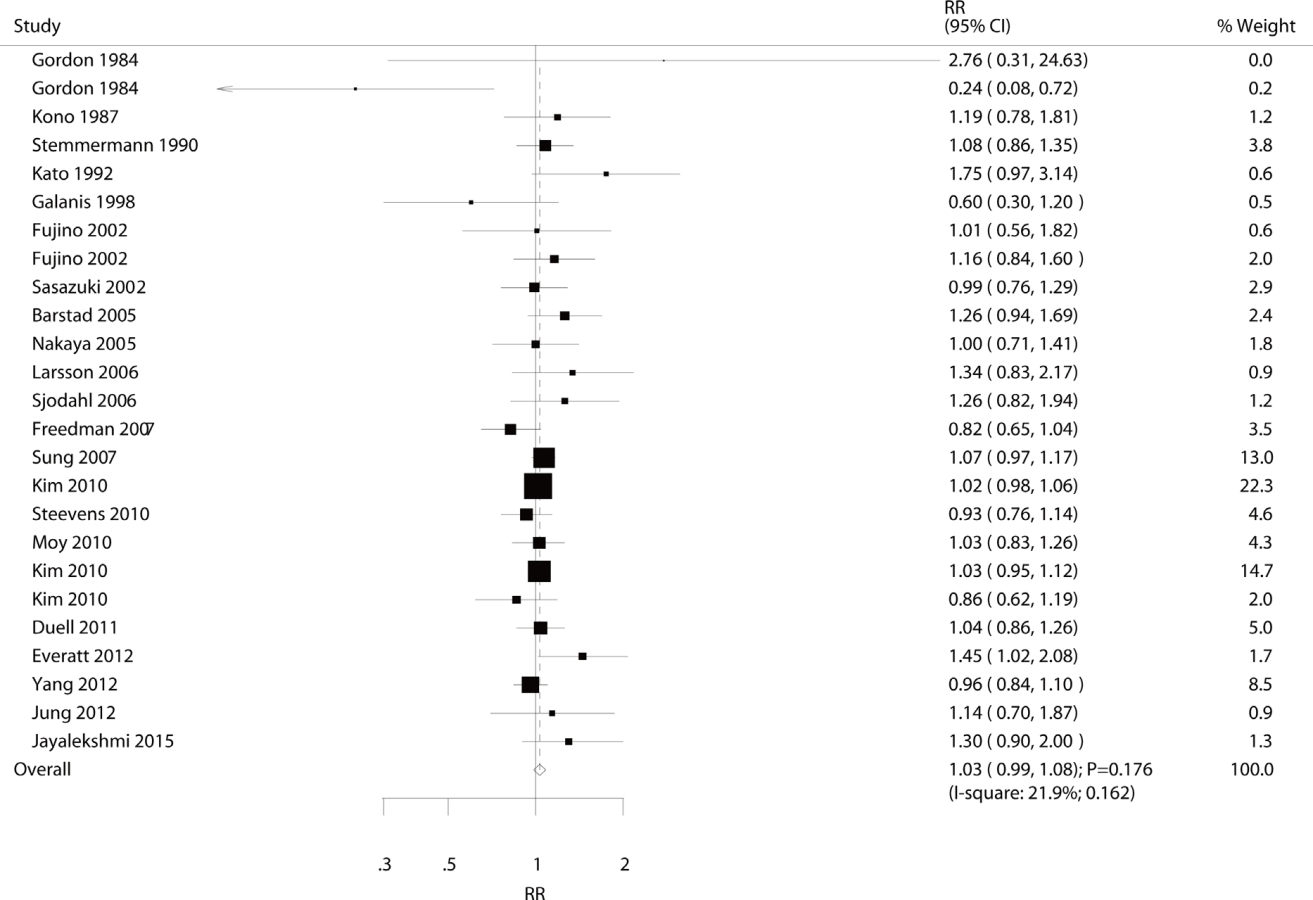
Association between drinkers versus non-drinkers and the risk of gastric cancer

A total of 22 studies reported an association between alcohol consumption and the risk of gastric cancer. The summary RR showed that drinking participants in general were not associated with a greater risk of gastric cancer (RR: 1.03; 95% CI: 0.99–1.08; *P* = 0.176; Figure 2), with moderate heterogeneity detected (I^2^ = 21.9%; *P* = 0.162). A sensitivity analysis indicated that the conclusion was not changed after sequential exclusion of each individual study (Table 2). Subgroup analysis indicated no significant associations between general alcohol consumption and the risk of gastric cancer in any specific subpopulations (Table 3).

**Table 2:** Sensitivity analysis for drinkers vs non-drinkers

Excluding study	RR and 95% CI	*P* value	Heterogeneity (%)	*P* value for heterogeneity
Gordon 1984	1.03 (0.98–1.08)	0.184	23.2	0.151
Gordon 1984	1.03 (1.00–1.07)	0.088	4.2	0.404
Kono 1987	1.03 (0.98–1.08)	0.208	24.0	0.142
Stemmermann 1990	1.03 (0.98–1.09)	0.217	24.7	0.134
Kato 1992	1.03 (0.98–1.08)	0.202	16.6	0.233
Galanis 1998	1.04 (0.99–1.08)	0.139	19.1	0.200
Fujino 2002	1.03 (0.98–1.09)	0.183	25.2	0.129
Fujino 2002	1.03 (0.98–1.08)	0.219	23.8	0.144
Sasazuki 2002	1.04 (0.98–1.09)	0.173	25.0	0.131
Barstad 2005	1.03 (0.98–1.08)	0.247	20.3	0.185
Nakaya 2005	1.03 (0.98–1.09)	0.179	25.1	0.130
Larsson 2006	1.03 (0.98–1.08)	0.212	22.2	0.163
Sjodahl 2006	1.03 (0.98–1.08)	0.214	23.0	0.153
Freedman 2007	1.04 (0.99–1.09)	0.084	15.3	0.249
Sung 2007	1.03 (0.98–1.09)	0.294	23.1	0.152
Kim 2010	1.04 (0.98–1.10)	0.210	24.5	0.136
Steevens 2010	1.04 (0.99–1.09)	0.130	22.8	0.155
Moy 2010	1.03 (0.98–1.09)	0.193	25.2	0.129
Kim 2010	1.04 (0.98–1.10)	0.219	25.2	0.130
Kim 2010	1.04 (0.99–1.09)	0.136	22.3	0.162
Duell 2011	1.03 (0.98–1.09)	0.201	25.1	0.130
Everatt 2012	1.03 (0.98–1.07)	0.232	15.2	0.251
Yang 2012	1.04 (0.99–1.10)	0.122	22.6	0.157
Jung 2012	1.03 (0.98–1.08)	0.197	24.7	0.134
Jayalekshmi 2015	1.03 (0.98–1.08)	0.222	21.7	0.168

**Table 3:** Subgroup analysis for drinkers versus non-drinkers and the risk of gastric cancer

Group	RR and 95% CI	*P* value	Heterogeneity (%)	*P* value for heterogeneity	Ratio between subgroups	*P* value for interaction test
Country						
US or Europe	1.04 (0.89–1.21)	0.620	54.5	0.015	1.01 (0.86–1.18)	0.904
Asia	1.03 (1.00–1.06)	0.090	0.0	0.791		
Sample size						
≥ 10000	1.03 (1.00–1.06)	0.098	0.0	0.704	0.97 (0.76–1.25)	0.821
< 10000	1.06 (0.83–1.36)	0.658	61.2	0.012		
Gender						
Men	1.04 (0.97–1.12)	0.230	28.1	0.161	1.03 (0.79–1.33)	0.825
Women	1.01 (0.79–1.30)	0.945	2.4	0.380		
Outcomes						
GC incidence	1.05 (0.99–1.11)	0.135	24.5	0.177	1.04 (0.92–1.17)	0.521
GC mortality	1.01 (0.91–1.12)	0t.919	24.6	0.225		
Adjusted BMI or not						
Yes	1.02 (0.99–1.06)	0.152	1.2	0.432	0.94 (0.81–1.08)	0.358
No	1.09 (0.95–1.25)	0.206	37.5	0.083		
Adjusted educational attainment						
Yes	1.02 (0.93–1.12)	0.707	24.3	0.205	0.98 (0.88–1.09)	0.722
No	1.04 (0.99–1.10)	0.127	23.8	0.203		
Adjusted physical activity						
Yes	1.00 (0.95–1.07)	0.897	30.9	0.227	0.93 (0.85–1.02)	0.151
No	1.07 (0.99–1.14)	0.072	19.6	0.206		

### Light alcohol consumption versus non-drinkers for gastric cancer

**Figure 3:**
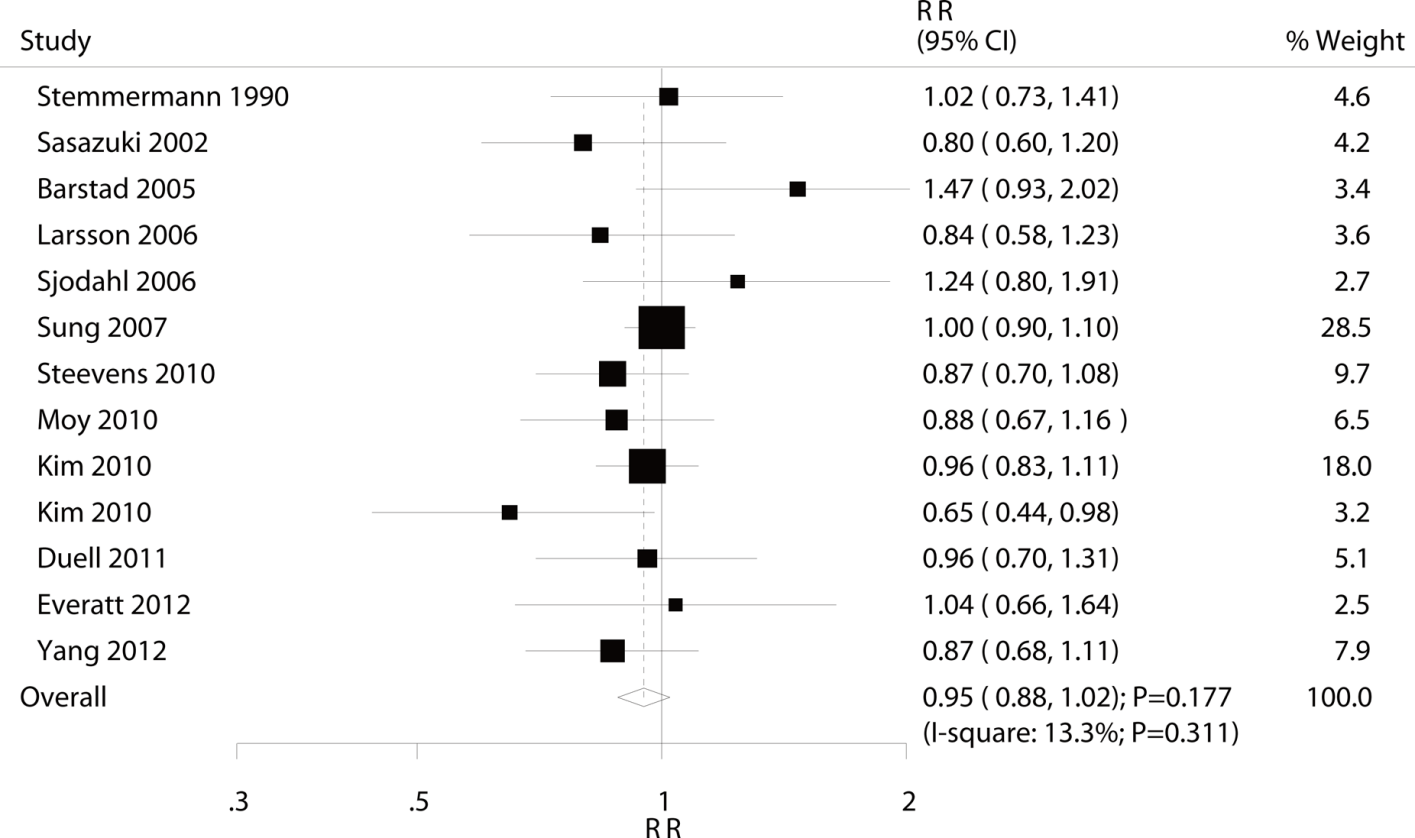
Association between light alcohol consumption and the risk of gastric cancer

A total of 12 studies reported an association between light alcohol consumption and the risk of gastric cancer. Pooled analysis results indicated that there was no association between alcohol consumption and gastric cancer (RR: 0.95; 95% CI: 0.88–1.02; *P* = 0.177; Figure 3). Furthermore, no significant heterogeneity was detected and the results of the sensitivity analysis indicated that the conclusion was not affected by the exclusion of any specific study (Supplementary Table 1). Subgroup analysis indicated that light alcohol consumption was associated with lower risk of gastric cancer in women (RR: 0.74; 95% CI: 0.57–0.98; *P* = 0.035; Table 4), whereas no other significant differences were detected.

**Table 4:** Subgroup analysis for light, moderate, and heavy drinking versus non-drinkers and the risk of gastric cancer

	Subgroup	Light alcohol	*P* value	Moderate alcohol	*P* value	Heavy alcohol	*P* value
Country	US or Europe	1.00 (0.87–1.15)	0.961	1.03 (0.87–1.22)	0.721	**1.20 (1.03–1.41)**	**0.021**
	Asia	0.93 (0.85–1.02)	0.108	1.06 (0.97–1.15)	0.177	**1.12 (1.04–1.20)**	**0.002**
	US or Europe vs Asia	1.08 (0.91–1.27)	0.393	0.97 (0.80–1.17)	0.766	1.07 (0.90–1.27)	0.433
Sample size	≥ 10000	0.95 (0.86–1.04)	0.270	1.07 (0.99–1.15)	0.101	**1.13 (1.06–1.21)**	**< 0.001**
	< 10000	0.93 (0.79–1.10)	0.393	0.92 (0.61–1.39)	0.686	1.23 (0.89–1.70)	0.214
	≥ 10000 vs < 10000	1.02 (0.84–1.24)	0.827	1.16 (0.77–1.77)	0.479	0.92 (0.66–1.28)	0.615
Gender	Men	0.96 (0.90–1.03)	0.302	1.06 (0.97–1.14)	0.188	**1.13 (1.06–1.22)**	**0.001**
	Women	**0.74 (0.57–0.98)**	0.035	1.30 (0.90–1.87)	0.156	1.33 (0.79–2.24)	0.285
	Men vs women	1.30 (0.98–1.72)	0.068	0.82 (0.56–1.19)	0.285	0.85 (0.50–1.44)	0.544
Reported	GC incidence	0.98 (0.90–1.06)	0.551	1.08 (0.99–1.17)	0.102	**1.18 (1.08–1.29)**	**< 0.001**
outcomes	GC mortality	0.87 (0.73–1.05)	0.141	1.01 (0.88–1.15)	0.942	1.07 (0.97–1.19)	0.170
	GC incidence vs GC mortality	1.13 (0.92–1.37)	0.242	1.07 (0.91–1.25)	0.405	1.10 (0.96–1.26)	0.157
Adjusted	Yes	0.95 (0.89–1.02)	0.173	1.07 (0.98–1.17)	0.119	**1.17 (1.08–1.26)**	**< 0.001**
BMI or not	No	1.00 (0.80–1.26)	0.985	0.98 (0.83–1.15)	0.781	1.05 (0.93–1.19)	0.440
	Yes vs no	0.95 (0.75–1.20)	0.672	1.09 (0.91–1.31)	0.353	1.11 (0.96–1.29)	0.145
Adjusted	Yes	0.91 (0.81–1.02)	0.106	1.02 (0.90–1.17)	0.722	1.10 (0.99–1.22)	0.070
educational	No	0.97 (0.84–1.10)	0.606	1.07 (0.98–1.18)	0.133	**1.16 (1.06–1.26)**	**0.001**
attainment	Yes vs no	0.94 (0.79–1.12)	0.480	0.95 (0.81–1.12)	0.559	0.95 (0.85–1.05)	0.327
Adjusted	Yes	0.83 (0.57–1.20)	0.317	1.04 (0.91–1.20)	0.558	1.13 (0.99–1.28)	0.070
physical	No	0.97 (0.90–1.04)	0.373	1.05 (0.97–1.15)	0.223	**1.14 (1.05–1.23)**	**0.001**
activity	Yes vs no	0.86 (0.59–1.25)	0.420	0.99 (0.84–1.17)	0.908	0.99 (0.85–1.15)	0.909

### Moderate alcohol consumption versus non-drinking for gastric cancer

**Figure 4:**
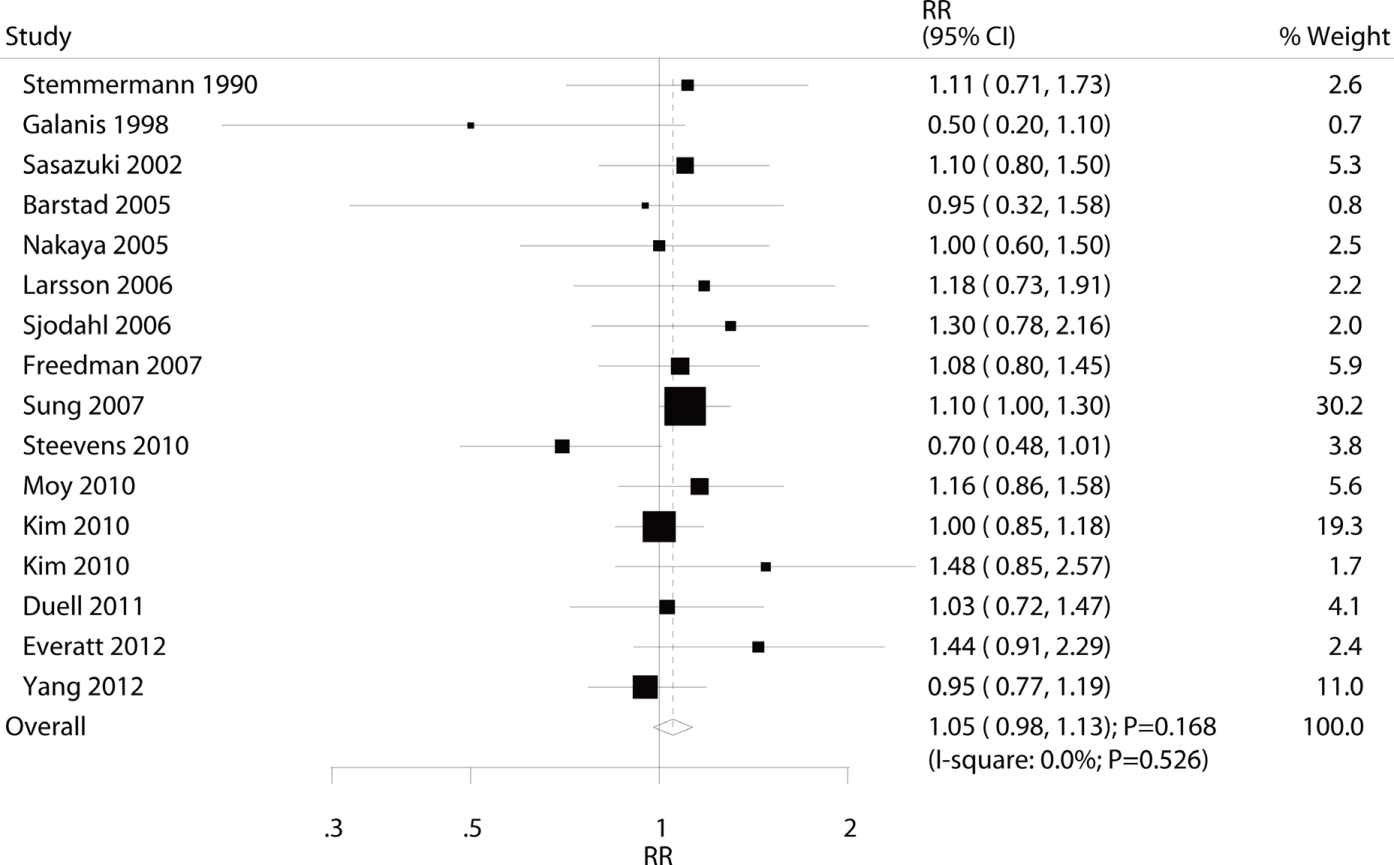
Association between moderate alcohol consumption and the risk of gastric cancer

A total of 15 studies reported an association between moderate alcohol consumption and the risk of gastric cancer. There was no significant association between moderate alcohol consumption and the risk of gastric cancer (RR: 1.05; 95% CI: 0.98–1.13; *P* = 0.168; with no evidence of heterogeneity. Figure 4). A sensitivity analysis was conducted, and the conclusion was not affected by the exclusion of any specific study from the pooled analysis (Supplementary Table 2). Subgroup analyses suggested no significant associations between moderate alcohol consumption and the risk of gastric cancer with any specific participant characteristics (Table 4).

### Heavy alcohol consumption versus non-drinking for gastric cancer

**Figure 5:**
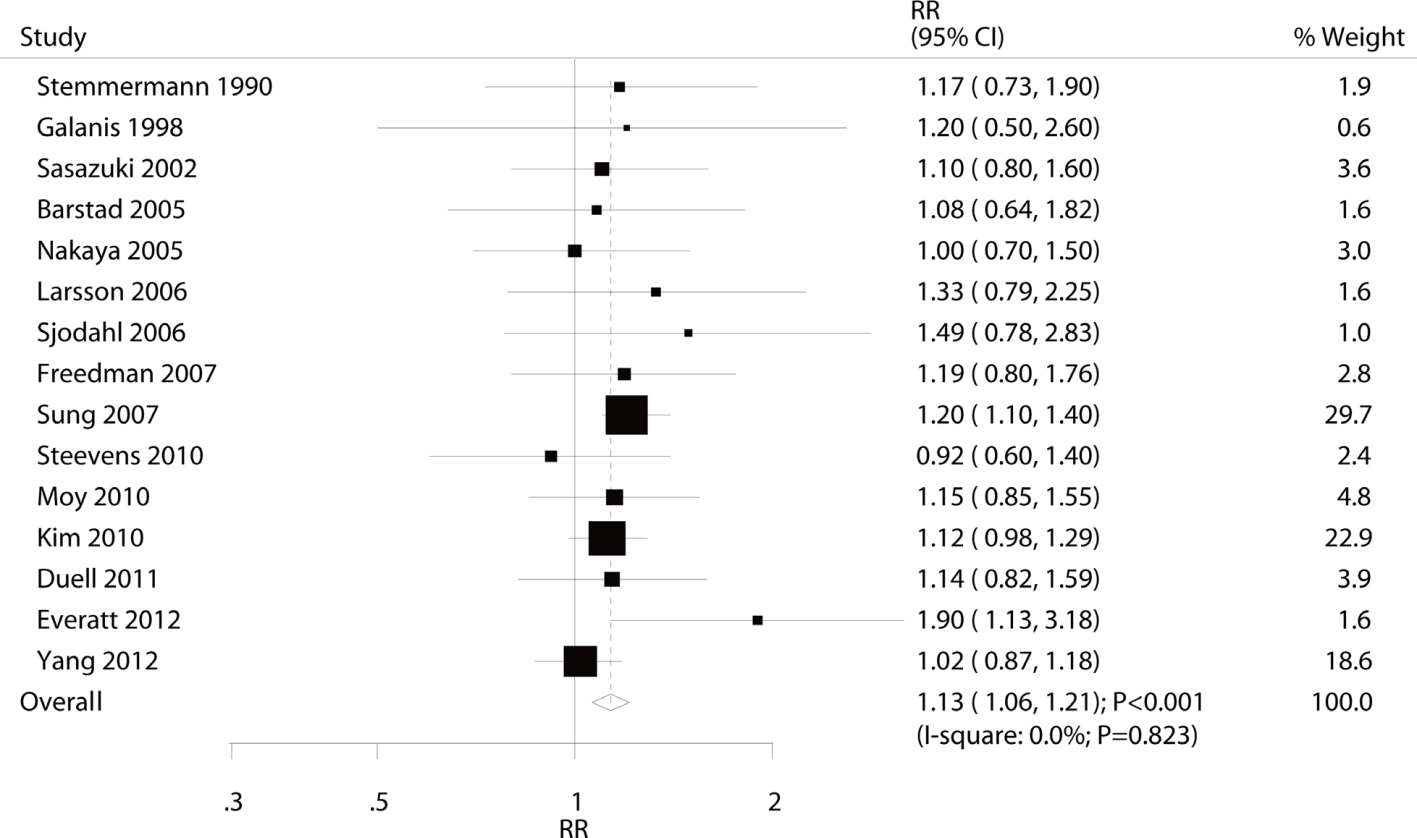
Association between heavy alcohol consumption and the risk of gastric cancer

A total of 15 studies reported an association between heavy alcohol consumption and the risk of gastric cancer. The pooled analysis results for gastric cancer indicated that the comparison of the heavy alcohol consumption versus non-drinking showed a harmful effect (RR: 1.13; 95% CI: 1.06–1.21; *P* < 0.001; without evidence of heterogeneity; Figure 5). The sensitivity analysis was conducted, and the conclusions were not changed by the exclusion of any individual study (Supplementary Table 3). Subgroup analyses indicated that heavy alcohol consumption was associated with an increased risk of gastric cancer regardless of country (US or Europe: RR, 1.20; 95% CI, 1.03–1.41, *P* = 0.021; Asia: RR, 1.12; 95% CI, 1.04–1.20, *P* = 0.002), whether the sample size of the study was ≥ 10,000 (RR: 1.13; 95% CI: 1.06–1.21; *P* < 0.001), whether participants were male (RR: 1.13; 95% CI: 1.06–1.22; *P* = 0.001), whether the study reported gastric cancer incidence (RR: 1.18; 95% CI: 1.08–1.29; *P* < 0.001), whether the study adjusted for BMI (RR: 1.17; 95% CI: 1.08-1.26; *P* < 0.001), whether the study adjusted for educational attainment (RR: 1.16; 95% CI: 1.06–1.26; *P* = 0.001), and whether the study adjusted for physical activity (RR: 1.14; 95% CI: 1.05–1.23; *P* = 0.001).

### Dose-response analysis

**Figure 6:**
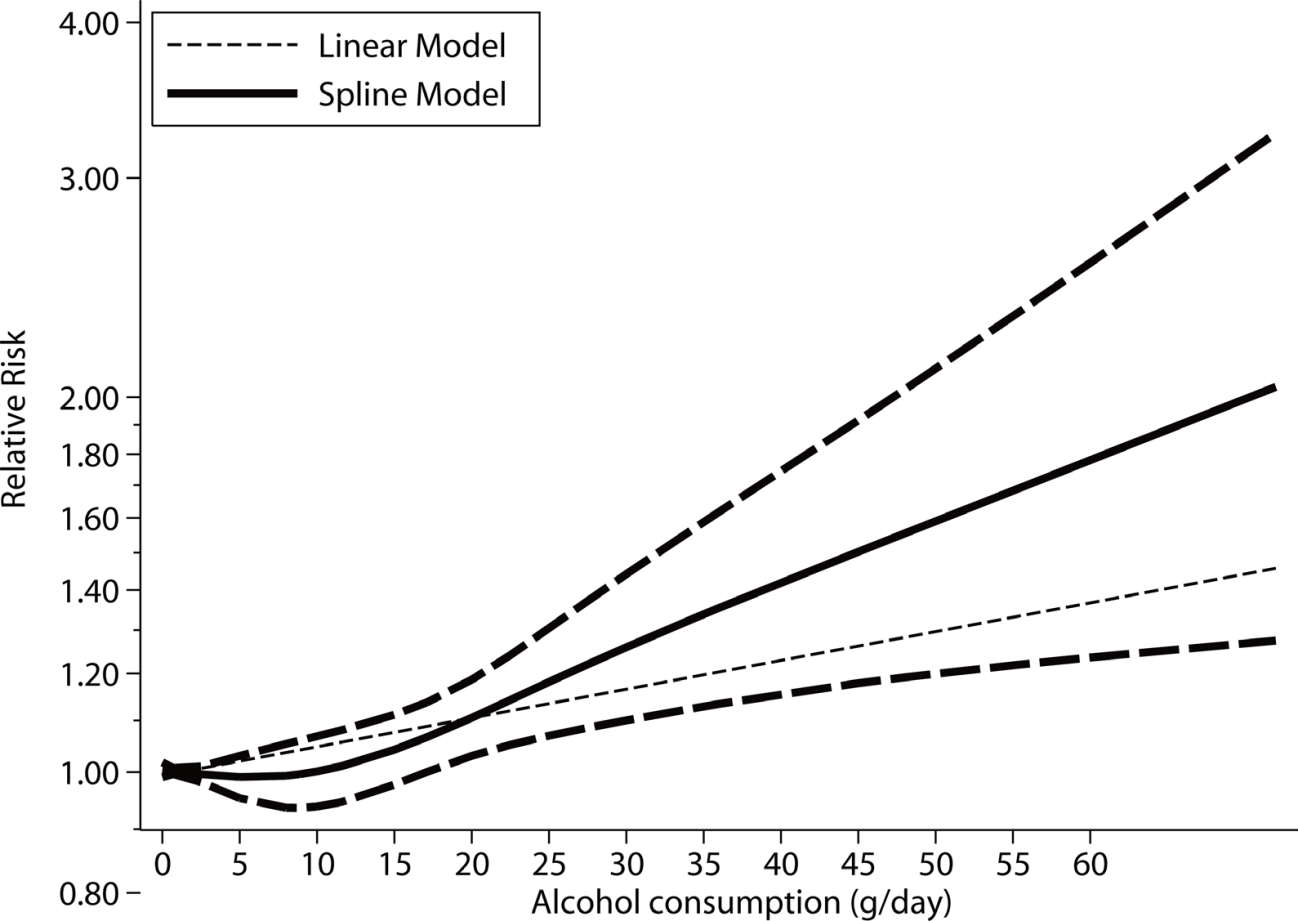
Dose-response relations between alcohol consumption and relative risks of gastric cancer

As shown by Figure 6 the *P* value for nonlinearity (*P* = 0.308), we found there was no statistically significant of nonlinear relationships between alcohol consumption intake and the risk of gastric cancer. Further, we noted all range of alcohol consumption were not associated with the risk of gastric cancer.

### Publication bias

**Figure 7:**
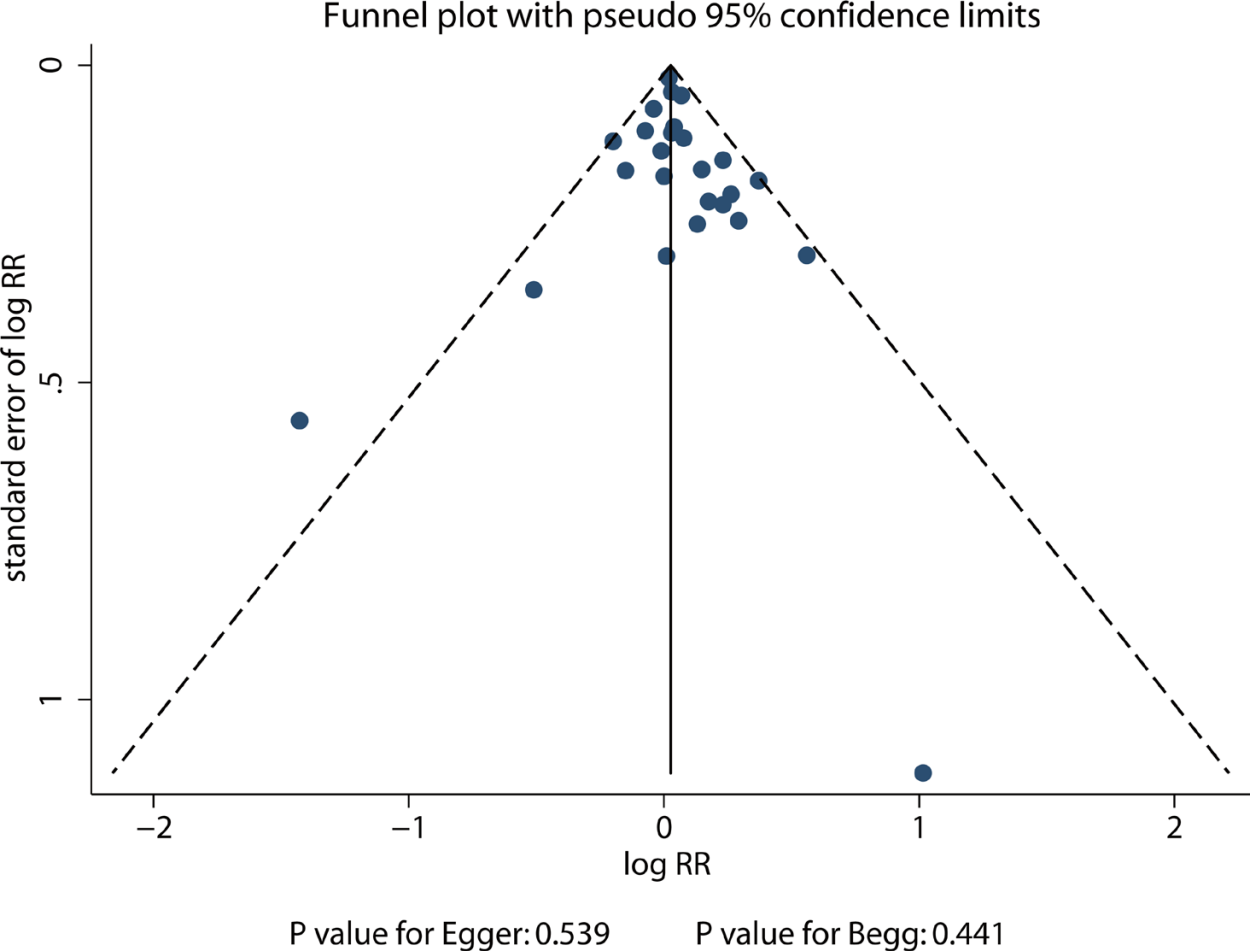
Funnel plot for drinkers versus non-drinkers and gastric cancer risk

A review of the funnel plots could not rule out the potential for publication bias for drinkers versus non-drinkers and gastric cancer risk (Figure 7). Furthermore, the Egger and Begg test results showed no evidence of publication bias (*P* value for Egger: 0.539; *P* value for Begg: 0.441).

## DISCUSSION

In the present meta-analysis, we reviewed twenty-two prospective cohort studies regarding the association between alcohol consumption and the risk of gastric cancer in 5,820,431 participants and 22,545 cases of gastric cancer morbidity or mortality with specific focus on broad characteristics. The pooled results concluded that there is a significant association between heavy alcohol consumption and gastric cancer with an overall 1.13-fold (95% CI: 1.06–1.21) increased risk in comparison with non-drinkers. Furthermore, light alcohol consumption might play a protective effect against gastric cancer in women.

A previous meta-analysis suggested that current drinkers with heavy alcohol consumption were associated with a greater risk of gastric cancer than non-drinker and indicated that the pooled RR was higher for gastric non-cardia than for gastric cardia adenocarcinomas, although these relationships did not have a statistically significant association [14]. Other studies posit that participants who consumed heavy alcohol also had an association with poor nutrition, which might affect the incidence of gastric cancer [39, 40]. However, since these findings were based on 44 case control and 15 cohort studies, the summary results and the findings of the stratified analysis might be biased due to the traditional case control study format introducing potential uncontrollable biases. Another meta-analysis based on 10 case control studies suggested that moderate and heavy alcohol consumption was associated with an elevated risk of gastric cancer, yet most studies were not included and the results are unreliable [15]. We therefore conducted this comprehensive quantitative meta-analysis of prospective cohort studies to better evaluate any possible association between alcohol consumption and gastric cancer.

The individual findings of most of the included studies agreed with the summary result for drinking versus non-drinking and gastric cancer risk. All included studies reported that light or moderate alcohol consumption had no significant effect on gastric cancer. However, the study conducted by Everatt et al reported an inconsistent result. This prospective study included 7,150 individuals in Lithuania and found that participants who consumed > 100 g/week ethanol had a 90% increased risk of gastric cancer versus 0.1–9.9 g ethanol/week [35]. A possible reason for this difference could be that this amount of alcohol intake was significantly higher than the cutoff for the heavy drinkers group used for this analysis, so this higher level could induce gastric cancer over the heavy alcohol consumption category reported here. Furthermore, the duration of the follow-up periods were greater than expected, allowing investigators to acquire a large number of gastric cancer cases and obtain narrow 95% CI, i.e., greater statistical significance.

The summary results suggested light and moderate alcohol consumption were not associated with the risk of gastric cancer, while several studies have reported inconsistent results. The study conducted by Kim et al suggested alcohol consumption play a positively association with the risk of gastric cancer mortality [33]. Further, increased alcohol consumption were associated with an increased risk of distal and total gastric cancer [29]. The possible reason for this could be ethanol involved in cancer development through acetaldehyde, which could enhanced pro-carcinogenic activity, modulation of cell regeneration, and nutritional deficiencies [33]. In addition, this analysis suggests that heavy alcohol consumption plays a harmful effect on gastric cancer. Although most included studies reported no association for this, two of included studies reported similar results: Sung et al indicated a positive relationship between alcohol consumption and distal or total gastric cancer risk [29], and Everatt et al demonstrated a positive link between alcohol consumption and gastric cancer incidence in the Lithuanian population [35]. A possible reason for this could be that a key metabolite acetaldehyde, which can have a local toxic effect and correlates with the incidence of gastric cancer [41]. The pathogenesis caused by ethanol on gastric mucosal damage was correlated with the balance of gastric mucosal defense and external invasion [28, 31, 42]. Finally, the amount of alcohol consumption was correlated to the role of aldehyde dehydrogenase and alcohol dehydrogenase polymorphisms, which have already been demonstrated to associate with the risk of head and neck, esophageal, and gastric cancers [43–45].

Subgroup analysis indicated that light alcohol consumption was associated with a reduction in gastric cancer in women. A possible reason for this relationship could entail how alcohol consumption might affect women who have received menopausal hormone therapy, which could reduce the risk of gastric cancer [46, 47]. In addition, heavy alcohol consumption was associated with a greater risk of gastric cancer in multiple subsets, which could be due to the stable relationship between heavy alcohol consumption and gastric cancer risk. These relationships in specific populations should be verified in further large-scale prospective cohort studies.

On dose-response meta-analysis, we found no nonlinear relationships between alcohol consumption and gastric cancer, and alcohol consumption has no significant effect on the risk of gastric cancer. The possible explanations for this could be that derive the dose-response curve required the distributions of cases and persons or person-years and effect estimate (RRs or HRs) with the variance estimates for at least 3 quantitative exposure categories, while these data were not available in several studies.

Four strengths of this meta-analysis should be highlighted, and are listed as follows: (1) our study was based on prospective cohort studies, which could eliminate selection and recall bias better than retrospective observational studies; (2) the alcohol consumption categories were divided into light, moderate, and heavy alcohol consumption, which could more accurately assess the dose relationship between alcohol consumption and gastric cancer; (3) a large number of participants was included, allowing us to quantitatively evaluate the association between alcohol consumption and the risk of gastric cancer morbidity and mortality, providing more robust findings for this study than those of any individual study; and (4) the RR were calculated in participants with specific characteristics to evaluate the difference in each subset for the relationship between alcohol consumption and the risk of gastric cancer.

The limitations of this study should also be acknowledged. First, the adjusted models are different across the included studies, and these factors might play an important role in the development of gastric cancer. Second, the range of alcohol consumption and cut-off values for consumption categories differed between studies, which might bias the observed effects of alcohol consumption. Third, *Helicobacter pylori* infection is a major risk factor for gastric cancer, but nearly all of the included studies were not adjusted for *H. pylori* status [48]. Fourth, the relationship between alcohol consumption and gastric cancer according to different histological type were not calculated due to smaller number of studies reported the effect estimate separately. Finally, this study was based on summary effect estimates, and individual data were not available, restricting us from conducting a more detailed relevant analysis.

The findings of this study suggest that, as compared with non-drinkers, drinking in general was not associated with the risk of gastric cancer. Light alcohol consumption might play an important role on gastric cancer in women, while no significant association was seen in other subsets. There was also no significant difference seen on gastric cancer risk for moderate alcohol consumption. Finally, heavy alcohol consumption significantly increased the risk of gastric cancer across all subgroups. Future studies should focus on specific population characteristics and different gastric cancer subtypes to further analyze this relationship.

## MATERIALS AND METHODS

### Data sources, search strategy, and selection criteria

This study was conducted and reported following the Meta-analysis of Observational Studies in Epidemiology (MOOSE) guidelines [49]. Three electronic databases (PubMed, Embase, and Cochrane Library) were searched from their dates of inception through April 2017 for prospective cohort studies published in English. The core search terms included (“alcohol” OR “ethanol” OR “alcoholic”) AND (“gastric” OR “stomach”) AND (“carcinoma” OR “cancer” OR “neoplasm” OR “adenocarcinoma”) AND “cohort” and excluded medical subject headings. Furthermore, the reference lists of the potentially included studies were reviewed to identify additional relevant studies. If multiple published reports from the same study were available, we included only the one with the most detailed information for both exposure and outcome. The medical subject heading, methods, patient population, design, exposure, and reported outcome were used to identify relevant studies.

The literature search and study selection was undertaken independently by 2 authors using a standardized approach. Any inconsistencies between these 2 authors were settled by group discussion until a consensus was reached. Studies that met the following criteria were included: (1) prospective cohort study with adult participants (i.e., 18 years or older); (2) reported outcomes of gastric cancer incidence or gastric cancer mortality; (3) the exposure of interest was alcohol consumption; and (4) reported adjusted risk estimates for the association between alcohol consumption and gastric cancer risk, or reported data sufficient to calculate these. Studies were excluded if they met the following exclusion criteria: (1) the study had a cross-sectional, case-control, retrospective cohort, or clinical trial design; (2) the study did not report effect estimates and 95% confidence intervals (CIs); (4) the exposure of interest was not alcohol consumption; and (5) the incidence of gastric cancer or gastric cancer mortality was not reported.

### Data collection and quality assessment

Data were extracted independently by two authors and reviewed by a third author. The following data were extracted: first author's name, publication year, country, sample size, age at baseline, number of men and women, number of gastric cancer incidence/death cases, follow-up duration periods, adjusted factors, and study design factors. A predesigned Excel (Microsoft Corporation) file was used to extract relevant information.

The Newcastle-Ottawa Scale (NOS) was used to evaluate methodological quality, which is quite comprehensive and has been partially validated for evaluating the quality of observational studies in meta-analyses [50]. The NOS is based on the following 3 subscales: selection of the study group (zero to four stars), quality of the adjustment for confounders (zero to two stars) and assessment of outcome or exposure (zero to three stars). A “star system” (range, 0–9) has been developed for assessment, with a higher score representing better methodological quality.

### Statistical analysis

We examined the relationship between alcohol consumption and risk of gastric cancer morbidity and mortality on the basis of the effect estimate (odds ratio [OR], relative risk [RR], or hazard ratio [HR]) and its 95% CI published in each study. A semi-parametric method was employed to evaluate the association between light (0–12 g per day), moderate (12–24 g per day), or heavy alcohol (≥ 24 g per day) consumption and the risk of gastric cancer morbidity and mortality. The value assigned to each alcohol consumption category was the mid-point for closed categories, and the median for open categories (assuming a normal distribution for alcohol consumption). Further, the alcohol consumption categories were consistent in men and women. In addition, the fixed effect model was employed to calculate the summary RRs and 95% confidence intervals (CIs) for each category of alcohol consumption if more than one median of alcohol consumption in each study was classified into light, moderate, and heavy alcohol consumption. We combined the relationship between the light, moderate, heavy alcohol consumption versus non-drinkers and the risk of gastric cancer morbidity and mortality by using the random-effects model [51, 52]. Further, the dose-response curve was derive based on restricted cubic splines with 3 knots at fixed percentiles of 10%, 50%, and 90% of the distribution [53, 54].

Heterogeneity between studies was investigated using the Q statistic, and we considered *P* values < 0.10 as indicative of significant heterogeneity [55, 56]. The sensitivity analyses were conducted for each category versus non-drinkers by removing each individual study from the overall analysis [57]. Subgroup analyses were conducted based on country, sample size, gender, reported outcomes, adjusted body mass index (BMI) or not, adjusted educational attainment or not, and adjusted physical activity or not. The ratio of RRs and the corresponding 95% CIs were calculated based on RRs and 95% CIs in each subset according to country, sample size, gender, reported outcomes, adjusted BMIR or not, adjusted educational attainment or not, and adjusted physical activity or not [58]. The funnel plot for drinkers versus non-drinkers and the risk of gastric cancer was performed, and the Egger [59] and Begg tests [60] were used to evaluate publication bias. All reported *P* values are 2-sided, and *P* values < 0.05 were considered statistically significant for all included studies. Statistical analyses were performed using STATA software (version 10.0; Stata Corporation, College Station, TX, USA).

## SUPPLEMENTARY MATERIALS FIGURES AND TABLES



## References

[1] Parkin DM, Läärä E, Muir CS (1988). Estimates of the worldwide frequency of sixteen major cancers in 1980. Inter J Cancer.

[2] Ferlay J, Soerjomataram I, Ervik M, Dikshit R, Eser S, Mathers C, Rebelo M, Parkin DM, Forman D, Bray F (2013). GLOBOCAN 2012 v1.0, Cancer Incidence and Mortality Worldwide: IARC CancerBase No. 11 [Internet].

[3] Bae JM, Kim EH (2016). Helicobacter pylori Infection and Risk of Gastric Cancer in Korea: A Quantitative Systematic Review. J Prev Med Public Health.

[4] Bae JM, Kim EH (2016). Dietary intakes of citrus fruit and risk of gastric cancer incidence: an adaptive meta-analysis of cohort studies. Epidemiol Health.

[5] Li L, Gan Y, Wu C, Qu X, Sun G, Lu Z (2015). Coffee consumption and the risk of gastric cancer: a meta-analysis of prospective cohort studies. BMC Cancer.

[6] Wu Y, Ye Y, Shi Y, Li P, Xu J, Chen K, Xu E, Yang J (2015). Association between vitamin A, retinol intake and blood retinol level and gastric cancer risk: Ameta-analysis. Clin Nutr.

[7] Chen Y, Yu C, Li Y (2014). Physical activity and risks of esophageal and gastric cancers: a meta-analysis. PLoS One.

[8] Uthman OA, Jadidi E, Moradi T (2013). Socioeconomic position and incidence of gastric cancer: a systematic review and meta-analysis. J Epidemiol Community Health.

[9] Bertuccio P, Rosato V, Andreano A, Ferraroni M, Decarli A, Edefonti V, La Vecchia C (2013). Dietary patterns and gastric cancer risk: a systematic review and meta-analysis. Ann Oncol.

[10] Yoon JM, Son KY, Eom CS, Durrance D, Park SM (2013). Pre-existing diabetes mellitus increases the risk of gastric cancer: a meta-analysis. World J Gastroenterol.

[11] Yang T, Yang X, Wang X, Wang Y, Song Z (2013). The role of tomato products and lycopene in the prevention of gastric cancer: a meta-analysis of epidemiologic studies. Med Hypotheses.

[12] Yu XF, Wang YQ, Zou J, Dong J (2012). A meta-analysis of the effects of energy intake on risk of digestive cancers. World J Gastroenterol.

[13] Bonequi P, Meneses-González F, Correa P, Rabkin CS, Camargo MC (2013). Risk factors for gastric cancer in Latin America: a meta-analysis. Cancer Causes Control.

[14] Tramacere I, Negri E, Pelucchi C, Bagnardi V, Rota M, Scotti L, Islami F, Corrao G, La Vecchia C, Boffetta P (2012). A meta-analysis on alcohol drinking and gastric cancer risk. Ann Oncol.

[15] Ma K, Baloch Z, He TT, Xia X (2017). Alcohol Consumption and Gastric Cancer Risk: A Meta-Analysis. Med Sci Monit.

[16] Estruch R, Sacanella E, Badia E, Antúnez E, Nicolás JM, Fernández-Solá J, Rotilio D, de Gaetano G, Rubin E, Urbano-Márquez A (2004). Different effects of red wine, gin consumption on inflammatory biomarkers of atherosclerosis: a prospective randomized crossover trial. Effects of wine on inflammatory markers. Atherosclerosis.

[17] Gordon T, Kannel WB (1984). Drinking and mortality. The Framingham Study. Am J Epidemiol.

[18] Kono S, Ikeda M, Tokudome S, Nishizumi M, Kuratsune M (1987). Cigarette smoking, alcohol and cancer mortality: a cohort study of male Japanese physicians. Jpn J Cancer Res.

[19] Stemmermann GN, Nomura AM, Chyou PH, Yoshizawa C (1990). Prospective study of alcohol intake and large bowel cancer. Dig Dis Sci.

[20] Kato I, Tominaga S, Matsumoto K (1992). A prospective study of stomach cancer among a rural Japanese population: a 6-year survey. Jpn J Cancer Res.

[21] Galanis DJ, Kolonel LN, Lee J, Nomura A (1998). Intakes of selected foods and beverages and the incidence of gastric cancer among the Japanese residents of Hawaii: a prospective study. Int J Epidemiol.

[22] Fujino Y, Tamakoshi A, Ohno Y, Mizoue T, Tokui N, Yoshimura T, JACC Study Group (2002). Japan Collaborative Cohort Study for Evaluation of Cancer Risk. Prospective study of educational background and stomach cancer in Japan. Prev Med.

[23] Sasazuki S, Sasaki S, Tsugane S (2002). Cigarette smoking, alcohol consumption and subsequent gastric cancer risk by subsite and histologic type. Int J Cancer.

[24] Barstad B, Sørensen TI, Tjønneland A, Johansen D, Becker U, Andersen IB, Grønbaek M (2005). Intake of wine, beer and spirits and risk of gastric cancer. Eur J Cancer Prev.

[25] Nakaya N, Tsubono Y, Kuriyama S, Hozawa A, Shimazu T, Kurashima K, Fukudo S, Shibuya D, Tsuji I (2005). Alcohol consumption and the risk of cancer in Japanese men: the Miyagi cohort study. Eur J Cancer Prev.

[26] Larsson SC, Giovannucci E, Wolk A (2006). Alcoholic beverage consumption and gastric cancer risk: a prospective population-based study in women. Int J Cancer.

[27] Sjödahl K, Lu Y, Nilsen TI, Ye W, Hveem K, Vatten L, Lagergren J (2006). Smoking and alcohol drinking in relation to risk of gastric cancer: a population-based, prospective cohort study. Int J Cancer.

[28] Freedman ND, Abnet CC, Leitzmann MF, Mouw T, Subar AF, Hollenbeck AR, Schatzkin A (2007). A prospective study of tobacco, alcohol, and the risk of esophageal and gastric cancer subtypes. Am J Epidemiol.

[29] Sung NY, Choi KS, Park EC, Park K, Lee SY, Lee AK, Choi IJ, Jung KW, Won YJ, Shin HR (2007). Smoking, alcohol and gastric cancer risk in Korean men: the National Health Insurance Corporation Study. Br J Cancer.

[30] Kim J, Park S, Nam BH (2010). Gastric cancer and salt preference: a population-based cohort study in Korea. Am J Clin Nutr.

[31] Steevens J, Schouten LJ, Goldbohm RA, van den Brandt PA (2010). Alcohol consumption, cigarette smoking and risk of subtypes of oesophageal and gastric cancer: a prospective cohort study. Gut.

[32] Moy KA, Fan Y, Wang R, Gao YT, Yu MC, Yuan JM (2010). Alcohol and Tobacco Use in Relation to Gastric Cancer: A Prospective Study of Men in Shanghai, China. Cancer Epidemiol Biomarkers Prev.

[33] Kim MK, Ko MJ, Han JT (2010). Alcohol consumption and mortality from all-cause and cancers among 1.34 million Koreans: the results from the Korea national health insurance corporation’s health examinee cohort in 2000. Cancer Causes Control.

[34] Duell EJ, Travier N, Lujan-Barroso L, Clavel-Chapelon F, Boutron-Ruault MC, Morois S, Palli D, Krogh V, Panico S, Tumino R, Sacerdote C, Quirós JR, Sánchez-Cantalejo E (2011). Alcohol consumption and gastric cancer risk in the European Prospective Investigation into Cancer and Nutrition (EPIC) cohort. Am J Clin Nutr.

[35] Everatt R, Tamosiunas A, Kuzmickiene I, Virviciute D, Radisauskas R, Reklaitiene R, Milinaviciene E (2012). Alcohol consumption and risk of gastric cancer: a cohort study of men in Kaunas, Lithuania, with up to 30 years follow-up. BMC Cancer.

[36] Yang L, Zhou M, Sherliker P, Cai Y, Peto R, Wang L, Millwood I, Smith M, Hu Y, Yang G, Chen Z (2012). Alcohol drinking and overall and cause-specific mortality in China: nationally representative prospective study of 220 000 men with 15 years of follow-up. Int J Epidemiol.

[37] Jung EJ, Shin A, Park SK, Ma SH, Cho IS, Park B, Lee EH, Chang SH, Shin HR, Kang D, Yoo KY (2012). Alcohol Consumption and Mortality in the Korean Multi-center Cancer Cohort Study. J Prev Med Public Health.

[38] Jayalekshmi PA, Hassani S, Nandakumar A, Koriyama C, Sebastian P, Akiba S (2015). Gastric cancer risk in relation to tobacco use and alcohol drinking in Kerala, India - Karunagappally cohort study. World J Gastroenterol.

[39] Stroup DF, Berlin JA, Morton SC, Olkin I, Williamson GD, Rennie D, Moher D, Becker BJ, Sipe TA, Thacker SB (2000). Meta-analysis of observational studies in epidemiology: a proposal for reporting. Meta-analysis Of Observational Studies in Epidemiology (MOOSE) group. JAMA.

[40] Stang A (2010). Critical evaluation of the Newcastle-Ottawa scale for the assessment of the quality of nonrandomized studies in meta-analyses. Eur J Epidemiol.

[41] DerSimonian R, Laird N (1986). Meta-analysis in clinical trials. Control Clin Trials.

[42] Ades AE, Lu G, Higgins JP (2005). The interpretation of random-effects meta-analysis in decision models. Med Decis Making.

[43] Deeks JJ, Higgins JPT, Altman DG, Higgins J, Green S (2008). Analyzing data and undertaking meta-analyses. Cochrane Handbook for Systematic Reviews of Interventions 5.0.1.

[44] Higgins JPT, Thompson SG, Deeks JJ, Altman DG (2003). Measuring inconsistency in meta-analyses. BMJ.

[45] Tobias A (1999). Assessing the influence of a single study in meta-analysis. Stata Tech Bull.

[46] Huxley RR, Woodward M (2011). Cigarette smoking as a risk factor for coronary heart disease in women compared with men: a systematic review and meta-analysis of prospective cohort studies. Lancet.

[47] Egger M, Davey Smith G, Schneider M, Minder C (1997). Bias in meta-analysis detected by a simple, graphical test. BMJ.

[48] Begg CB, Mazumdar M (1994). Operating characteristics of a rank correlation test for publication bias. Biometrics.

[49] D’Avanzo B, La Vecchia C, Braga C, Franceschi S, Negri E, Parpinel M (1997). Nutrient intake according to education, smoking, and alcohol in Italian women. Nutr Cancer.

[50] Klatsky AL (2001). Diet, alcohol, and health: a story of connections, confounders, and cofactors. Am J Clin Nutr.

[51] Bao P, Tao M, Liu D, Gao L, Jin F (2001). A case-control study of smoking, alcohol con-sumption and the occurrence of stomach cancer. Tumor.

[52] Wu AH, Wan P, Bernstein L (2001). A multiethnic population-based study of smok¬ing, alcohol and body size and risk of adenocarcinomas of the stomach an¬desophagus (United States). Cancer Causes Control.

[53] Hiraki A, Matsuo K, Wakai K, Suzuki T, Hasegawa Y, Tajima K (2007). Gene-gene and gene-environment interactions between alcohol drinking habit and polymorphisms in alcohol-metabolizing enzyme genes and the risk of head and neck cancer in Japan. Cancer Sci.

[54] Matsuo K, Hamajima N, Shinoda M, Hatooka S, Inoue M, Takezaki T, Tajima K (2001). Gene-environment interaction between an aldehyde dehydrogenase-2 (ALDH2) polymorphism and alcohol consumption for the risk of esophageal cancer. Carcinogenesis.

[55] Yokoyama A, Muramatsu T, Omori T, Yokoyama T, Matsushita S, Higuchi S, Maruyama K, Ishii H (2001). Alcohol and aldehyde dehydrogenase gene polymorphisms and oropharyngolaryngeal, esophageal and stomach cancers in Japanese alcoholics. Carcinogenesis.

[56] Green J, Czanner G, Reeves G, Watson J, Wise L, Roddam A, Beral V (2012). Menopausal hormone therapy and risk of gastrointestinal cancer: nested case-control study within a prospective cohort, and meta-analysis. Int J Cancer.

[57] Camargo MC, Goto Y, Zabaleta J, Morgan DR, Correa P, Rabkin CS (2012). Sex hormones, hormonal interventions, and gastric cancer risk: a meta-analysis. Cancer Epidemiol Biomarkers Prev.

[58] Munoz N, Franceschi S (1997). Epidemiology of gastric cancer and perspectives for prevention. Salud Publica Mex.

[59] Egger M, Davey Smith G, Schneider M, Minder C (1997). Bias in meta-analysis detected by a simple, graphical test. BMJ.

[60] Begg CB, Mazumdar M (1994). Operating characteristics of a rank correlation test for publication bias. Biometrics.

