# Supplementary material for: Association between alcohol consumption and the risk of gastric cancer: a meta-analysis of prospective cohort studies
Source: Oncotarget. 2017 Sep 14;8(48):84459–72. doi: 10.18632/oncotarget.20880 (PMC5663611; doi:10.18632/oncotarget.20880)
Supplement: Supplementary file 1 [file oncotarget-08-84459-s001.pdf]

Association between alcohol consumption and the risk of gastric cancer: a meta-analysis of prospective cohort studies

SUPPLEMENTARY MATERIALS

Supplementary Table 1: Sensitivity analysis for light alcohol consumption vs non-drinker

Excluding study	RR and 95% CI	*P* value	Heterogeneity (%)	*P* value for heterogeneity
Stemmermann 1990	0.94 (0.87–1.02)	0.166	19.6	0.251
Sasazuki 2002	0.96 (0.89–1.03)	0.262	13.8	0.309
Barstad 2005	0.95 (0.89–1.01)	0.091	0.0	0.620
Larsson 2006	0.95 (0.88–1.03)	0.235	17.6	0.271
Sjodahl 2006	0.94 (0.88–1.02)	0.126	11.7	0.331
Sung 2007	0.93 (0.85–1.02)	0.121	13.2	0.315
Steevens 2010	0.96 (0.89–1.04)	0.300	15.5	0.293
Moy 2010	0.95 (0.88–1.03)	0.250	18.2	0.265
Kim 2010	0.94 (0.86–1.03)	0.213	20.5	0.242
Kim 2010	0.97 (0.91–1.03)	0.300	0.0	0.517
Duell 2011	0.95 (0.87–1.03)	0.193	20.5	0.242
Everatt 2012	0.95 (0.87–1.02)	0.170	19.7	0.250
Yang 2012	0.96 (0.88–1.04)	0.275	16.7	0.280

Supplementary Table 2: Sensitivity analysis for moderate alcohol consumption vs non–drinker

Excluding study	RR and 95% CI	*P* value	Heterogeneity (%)	*P* value for heterogeneity
Stemmermann 1990	1.05 (0.98–1.13)	0.186	0.0	0.454
Galanis 1998	1.06 (0.98–1.14)	0.129	0.0	0.682
Sasazuki 2002	1.05 (0.97–1.13)	0.202	0.0	0.456
Barstad 2005	1.05 (0.98–1.13)	0.163	0.0	0.455
Nakaya 2005	1.05 (0.98–1.13)	0.163	0.0	0.454
Larsson 2006	1.05 (0.98–1.13)	0.196	0.0	0.467
Sjodahl 2006	1.05 (0.97–1.13)	0.212	0.0	0.502
Freedman 2007	1.05 (0.98–1.13)	0.196	0.0	0.453
Sung 2007	1.03 (0.95–1.12)	0.475	0.0	0.499
Steevens 2010	1.07 (0.99–1.15)	0.076	0.0	0.818
Moy 2010	1.05 (0.97–1.13)	0.236	0.0	0.482
Kim 2010	1.06 (0.98–1.15)	0.125	0.0	0.485
Kim 2010	1.05 (0.97–1.12)	0.227	0.0	0.566
Duell 2011	1.05 (0.98–1.13)	0.169	0.0	0.451
Everatt 2012	1.04 (0.97–1.12)	0.250	0.0	0.593
Yang 2012	1.07 (0.99–1.15)	0.105	0.0	0.523

Supplementary Table 3: Sensitivity analysis for heavy alcohol consumption vs non–drinker

Excluding study	RR and 95% CI	*P* value	Heterogeneity (%)	*P* value for heterogeneity
Stemmermann 1990	1.13 (1.06–1.21)	< 0.001	0.0	0.765
Galanis 1998	1.13 (1.06–1.21)	< 0.001	0.0	0.765
Sasazuki 2002	1.14 (1.06–1.21)	< 0.001	0.0	0.766
Barstad 2005	1.14 (1.06–1.21)	< 0.001	0.0	0.766
Nakaya 2005	1.14 (1.07–1.22)	< 0.001	0.0	0.796
Larsson 2006	1.13 (1.06–1.21)	< 0.001	0.0	0.791
Sjodahl 2006	1.13 (1.06–1.21)	< 0.001	0.0	0.815
Freedman 2007	1.13 (1.06–1.21)	< 0.001	0.0	0.768
Sung 2007	1.11 (1.02–1.20)	0.010	0.0	0.848
Steevens 2010	1.14 (1.07–1.22)	< 0.001	0.0	0.833
Moy 2010	1.13 (1.06–1.21)	< 0.001	0.0	0.764
Kim 2010	1.14 (1.06–1.23)	0.001	0.0	0.767
Duell 2011	1.13 (1.06–1.21)	< 0.001	0.0	0.764
Everatt 2012	1.12 (1.05–1.20)	< 0.001	0.0	0.969
Yang 2012	1.16 (1.08–1.25)	< 0.001	0.0	0.911
